# Effectiveness of a Dyadic Pain Management Program for Community-Dwelling Older Adults with Chronic Pain: A Cluster Randomized Controlled Trial

**DOI:** 10.3390/healthcare14040553

**Published:** 2026-02-23

**Authors:** Mimi Mun Yee Tse, Shamay Sheung Mei Ng, Paul H. Lee, Angel Shuk Kwan Tang, Percy Poo-see Tse, Kin Pong To, Sukki Ho, Timothy Chung Ming Wu

**Affiliations:** 1 School of Nursing and Health Sciences, Hong Kong Metropolitan University, Hong Kong, China; pptse@hkmu.edu.hk (P.P.-s.T.); kpto@hkmu.edu.hk (K.P.T.); tcmwu@hkmu.edu.hk (T.C.M.W.); 2 Department of Rehabilitation Sciences, The Hong Kong Polytechnic University, Hong Kong, China; shamay.ng@polyu.edu.hk; 3 Faculty of Medicine, University of Southampton, Southampton SO17 1BJ, UK; paul.h.lee@southampton.ac.uk; 4 School of Nursing, Caritas Medical Center, Hospital Authority, Hong Kong, China; sk.tang@ha.org.hk; 5 Faculty of Health and Social Sciences, The Hong Kong Polytechnic University, Hong Kong, China; sukki.ho@polyu.edu.hk

**Keywords:** older adults, non-pharmacological methods, pain management, chronic pain, digital health

## Abstract

**Background:** The demand for digitally supported chronic pain management has grown. Yet, the employment of a well-structured and sustainable program for older adults is limited due to insufficient research studies involving both older adults and their informal caregivers. **Objective:** This cluster randomized controlled trial evaluated the effectiveness of a Dyadic Pain Management (DPM) program, with the primary outcome of pain intensity. Secondary outcomes included pain interference, pain self-efficacy, activities of daily living, pain knowledge, psychological symptoms (depression, anxiety, stress), and caregiver burden. **Methods:** A cluster randomized controlled trial was conducted with 150 dyads (community-dwelling older adults with chronic non-cancer pain and their informal caregivers) over 8 weeks. The intervention comprised 4 weeks of on-campus group sessions followed by 4 weeks of WhatsApp-based support, while the control group received lesson pamphlets. Outcomes were assessed at baseline (T0), week 8 (T1), and week 16 (T2). **Results:** Statistically significant improvements in pain outcomes were observed in the intervention group compared with the control group over follow-up. Between-group differences were significant for pain intensity (primary outcome) and pain interference, and pain self-efficacy also improved. Significant between-group differences were also observed for depression, anxiety, and stress after the intervention, and caregiver burden was lower in the intervention group at follow-up. **Conclusions:** These findings suggest that a dyadic, non-pharmacological pain management program with a WhatsApp-based component may support improvements in pain- and psychosocial-related outcomes among community-dwelling older adults with chronic pain and their informal caregivers.

## 1. Introduction

Chronic pain is defined as “pain that persists or recurs for longer than 3 months,” and the International Association for the Study of Pain (IASP) further explained that chronic pain is linked to biological, psychological, and social factors [1]. Chronic pain is highly prevalent among community-dwelling older adults, approximating to be as high as 85% [2]. In Hong Kong alone, around 35% of older adults reported to experience chronic pain [3]. It substantially contributes to physical and psychosocial disabilities, mental distress, other noncommunicable diseases such as diabetes and hypertension, and lower quality of life. The burden of chronic pain and other related adverse outcomes continues to grow as the aging population increases [4,5]. Thus, addressing this global burden and enhancing the quality of life for older adults suffering from chronic pain is a public health priority.

Pain management involves a variety of diverse treatment options to help older adults manage their pain. Previous studies have examined the effectiveness of pharmacological and non-pharmacological methods on older adults in managing chronic pain and found a positive effect [5,6,7,8,9]. When comparing these two methods, non-pharmacological methods are preferable because they are less limited by side effects [10]. The complexity and uncertainty of traditional or drug pain interventions also hinders older adults from participating in these studies. Since polypharmacy (the practice of using several medications concurrently) is an extremely common phenomenon among older adults, several studies have recommended more exploration and frequent use of non-pharmacological methods for older adults [7,8,11].

With the surge of technological advancements, digitally supported interventions have become popular and transformed the landscape of randomized controlled trials (RCT) and participant engagement. Researchers can collect data via mobile applications (e.g., WhatsApp) and devices with real-time monitoring functions, ensuring the sufficient quantity and quality of data. When parts of the intervention are virtually delivered, participants with physical disabilities can engage in an RCT remotely via web-based/mobile conferencing platforms (e.g., Zoom), making it more convenient, less time-consuming, and costly. Also, researchers can provide more clear and real-time instructions via group chats formed in these mobile applications, encouraging more active participations, and fostering a friendly social environment. Thus, increasing more adherence to exercise [12,13]. Moreover, the Hong Kong government offered remote services to older adults during the coronavirus disease 2019 (COVID-19) pandemic. Several community centers transitioned their events to a digital format, while certain healthcare providers began providing telemedicine services to older adults [14]. Also, a systematic literature review highlighted the significance and effectiveness of digital tools in supporting older adults when they are given sufficient motivation and support [15]. Overall, remote interventions benefit researchers and older adults in terms of data collection, accessibility, flexibility, and cost-effectiveness.

Pender’s Health Promotion Model is a widely established framework that pinpoints the factors influencing health behaviors. These factors include self-efficacy, perceived benefits, surrounding cues, barriers, stress, environmental contributions, and more [16]. Several recent studies have adopted this model to evaluate and encourage health-promoting behaviors among their targeted populations and found it compelling [17,18,19,20]. This model has been well accepted in research, implying that health promotion framework-based interventions are highly effective.

The research team, including registered nurses, physiotherapist, social worker, professors and research assistants, applied the elements of Pender’s Health Promotion Model to the study’s intervention program because this model had gained substantial acceptance for investigating various health promoting behaviors and had demonstrated positive outcomes. Specially, we employed Pender’s Health Promotion Model to develop a dyadic intervention that leverages social support from an informal caregiver to strengthen older adults’ self-management of chronic pain, with additional digital reinforcement delivered via WhatsApp (e.g., reminders and program materials such as exercise videos) for both older adults and caregivers. Although digital health approaches are increasingly used, strategies to improve older adults’ ability and sustained engagement in self-pain management through accessible tools remain underexplored.

Accordingly, we formulated the following hypotheses: (H1—primary) compared with the control condition (receipt of pain-management pamphlets), participants receiving the Dyadic Pain Management (DPM) program will report a greater reduction in pain intensity over time. Secondary hypotheses were that, compared with controls, the DPM group will demonstrate greater improvements in pain interference and pain self-efficacy, better physical functioning/activities of daily living and pain-related knowledge, reduced psychological distress (depression, anxiety, stress), and reduced caregiver burden. We also developed a dedicated website on pain management, aiming to educate and inspire older adults to stay active [21].

In the present study, the digital component (WhatsApp) was conceptualized as an integral delivery mode within the multicomponent Dyadic Pain Management (DPM) program rather than a stand-alone intervention. Specifically, WhatsApp was designed to extend and reinforce the face-to-face content by providing ongoing “cues to action” (reminders and check-ins), easy access to program materials (e.g., exercise videos), and a low-barrier channel for dyadic engagement between older adults and their informal caregivers outside scheduled sessions. This digital add-on was therefore intended primarily to enhance adherence and sustained engagement in self-pain management practices taught during the in-person sessions, particularly when in-person contact was constrained. Accordingly, the trial evaluates the effectiveness of the combined in-person plus WhatsApp-supported DPM package, and the contribution of individual components (digital vs. face-to-face) should be interpreted as part of the overall program effect.

## 2. Methods

### 2.1. Sample Size

Our pilot study has demonstrated a significant reduction in pain scores within the intervention group, with a large effect size (Cohen’s *d* > 0.9) [22]. Thus, the sample size of this cluster RCT was calculated based on an effect size of 0.762 (= 1.6/2.1) on the pain score from our pilot study, with 80% power and a significance level of 5%. This estimation also accounted for an anticipated 20% attrition rate and an assumed intra-cluster correlation coefficient (ICC) of 0.10 to adjust the required sample size for clustering in this cluster randomized controlled trial [17]. Finally, we arrived at a sample size of 150 dyads in total, with 75 dyads in the experimental group and another 75 dyads in the control group.

### 2.2. Recruitment Process

The research team recruited 150 dyads of participants from the Neighborhood Elderly Centers (NECs) in the span of three years, where one older adult and one informal caregiver formed a dyad. Seventy-five dyads were randomly selected to receive the Dyadic Pain Management (DPM) intervention as the experimental group, and the other 75 dyads received pamphlets on managing pain as the control group. The randomization was conducted at the beginning of the study by a statistician independent of the research team, using a random numbers table to make the group assignments (1 = experimental DPM; 2 = control). After the randomization, the research team assigned each participant (older adult) into either the experimental group or control group. The informal caregivers followed the group assignments of the older adult participants, unknowingly. This randomization method could prevent any possible contamination effect.

#### 2.2.1. Inclusion Criteria

Older adults/Participants:

- Sixty-year-old or above who mainly received care from informal caregivers.
- Cantonese speaker.
- Achieved a score of more than 6 on the Abbreviated Mental Test, which is a valid threshold for distinguishing between normal and abnormal cognitive functions in geriatric clients [23].
- Experienced non-cancer related pain within the last six months [24].
- Reported a pain intensity of 2 or higher on the Numeric Rating Scale (a scale ranging from 0 to 11) [25].
  - Informal caregivers were eligible if they had experienced non-cancer-related pain within the last six months and reported a pain intensity of ≥2 on the Numeric Rating Scale (0–11). This criterion was used to ensure that both members of the dyad could actively participate in the shared pain self-management and exercise components and to enable evaluation of intervention-related changes in caregiver outcomes; however, we acknowledge that it may limit the generalizability of findings to caregiving populations without pain.
- Can participate in mild physical activities and stretching exercises.
- Possesses a smartphone with internet connectivity.

Informal caregivers:

- Eighteen-year-old or above.
- Informal caregiver of the participating older adults.
- Cantonese speaker.
- Achieved a score of more than 6 on the Abbreviated Mental Test, which is a valid threshold for distinguishing between normal and abnormal cognitive functions in geriatric clients [23].
- Experienced non-cancer related pain within the last six months [24].
- Reported a pain intensity of 2 or higher on the Numeric Rating Scale (a scale ranging from 0 to 11) [25].
- Can participate in mild physical activities and stretching exercises.
- Possesses a smartphone with internet connectivity.

#### 2.2.2. Exclusion Criteria

Older adults/Participants:

- Has significant visual and/or auditory impairments that hinder sight and hearing.
- Suffers from a serious organic illness or malignant tumor.
- Diagnosed with a mental disorder by a neurologist or psychiatrist.
- Underwent surgical procedures within the previous two months.
- Has a history of substance abuse.

Informal caregivers:

- Suffers from a serious organic illness or malignant tumor.
- Diagnosed with a mental disorder by a neurologist or psychiatrist.
- Underwent surgical procedures within the previous two months.
- Has a history of substance abuse.

### 2.3. Study Design

This current study presents the findings of a Dyadic Pain Management program (DPM), a cluster randomized controlled trial with a parallel group design. Participants (older adults and their informal caregivers) were randomly allocated in a 1:1 ratio into either the experimental group (*n* = 75 dyads) or the control group (*n* = 75 dyads). During the COVID-19 pandemic, the research team conducted a pilot study with 60 subjects where 30 subjects were placed in the experimental group and another 30 in the control group [26]. The pilot study demonstrated the feasibility and efficacy of the Pender’s Health Promotion Model-based DPM program.

In this cluster randomized controlled trial, randomization was performed at the cluster level, with Neighborhood Elderly Centers (NECs) serving as the clusters to minimize contamination between groups. A total of 20 NECs were initially contacted, and recruitment occurred across 18 participating centers over three years, resulting in an average of approximately 8 dyads per cluster (range: 5–12). Cluster assignments were generated by an independent statistician using a random numbers table (1 = experimental DPM group; 2 = control group), ensuring balanced allocation. Sample size calculations incorporated an assumed intra-cluster correlation coefficient (ICC) of 0.10, alongside an effect size of 0.762, 80% power, 5% significance level, and 20% attrition rate, yielding the target of 150 dyads. All statistical analyses fully accounted for the clustered structure at baseline (T0), week 8 (T1), and week 16 (T2) using multilevel regression models for normally distributed outcomes and generalized estimating equations (GEE) for non-normal data. These approaches nested observations within participants (level 1) and participants within NECs (level 2), explicitly modeling both within-subject correlations over time and intra-cluster correlations to provide unbiased estimates and appropriate standard errors.

**Figure 1:**
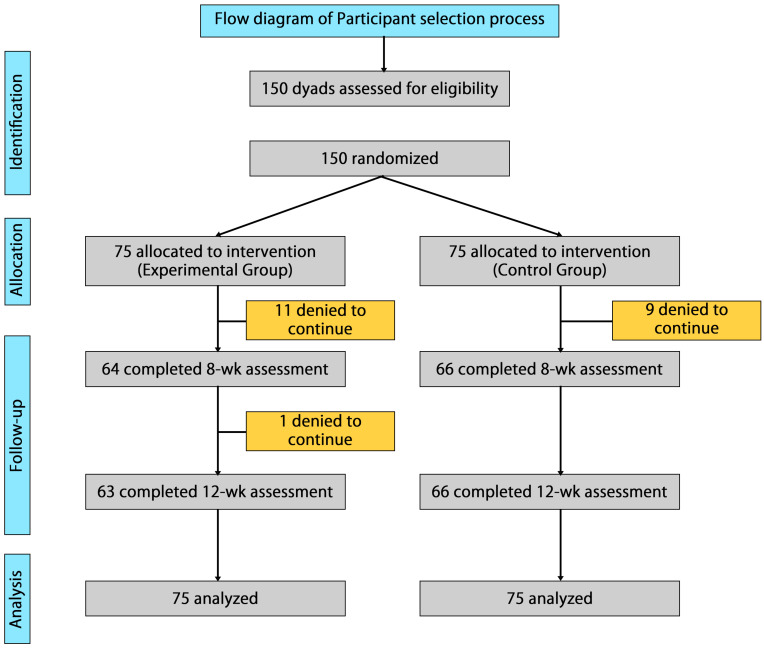
Participant flow diagram.

Figure 1 presents participant flow diagram showing identification, allocation, follow-up, and analysis stages of this study. This diagram illustrates the number of participants at each stage, including those withdrawn, in the cluster RCT conducted from June 2022 to December 2023.

### 2.4. Intervention—The Dyadic Pain Management Program (DPM)

The dyadic pain management program (DPM): a program that spanned over 8 weeks and was conducted in a group setting. The first half of the DPM, which lasted for 4 weeks, involved activities completed in person on the university campus. The remaining 4 weeks of the program comprised activities conducted digitally via a WhatsApp group. The research team arranged some timely make-up sessions for participants who missed the scheduled sessions.

In the on-campus (face-to-face) segment of the DPM, each session began with a 20 to 30 min of physical exercise routine, overseen by a research assistant. This was then followed by a 20 min educational segment on managing pain, which covered the effects of pain, the application of pharmacological and non-pharmacological strategies for managing pain, and practical demonstrations of various non-pharmacological pain management techniques. The research team instructed participants and their informal caregivers on communication skills related to the execution of various pain management techniques. The research team encouraged participants and their informal caregivers to practice these pain alleviation methods at home.

In the digital segment of the DPM, the research team added the participants and their informal caregivers to a WhatsApp group chat where they received the educational materials and video demonstrations of the physical exercises taught during the on-campus sessions. The research team sent reminders in the group chat to ensure participants and their informal caregivers carry out the 30 min exercise together at home thrice a week. They were also asked to log their use of different non-pharmacological pain relief methods and their perceived effectiveness of these methods in the WhatsApp group chat.

We will evaluate the digital component using prespecified engagement indicators, including WhatsApp message frequency (sent/received per dyad per week), responsiveness, and duration of active participation (weeks with ≥1 interaction). Adherence to home exercises will be assessed via exercise logs/app checklist/weekly self-report, summarized as sessions completed per week and proportion meeting the prescribed dose. Engagement will also be examined for variability across dyads (median, IQR; and distribution plots), enabling assessment of whether outcomes differ by level of WhatsApp participation.

**Trial Registration:** ClinicalTrials.gov NCT05056623 (Registration date: 24 March 2021).

### 2.5. Data Collection

Data collection was carried out from June 2022 to December 2023. Data was collected at three time points: At baseline (T0), week 8 (T1), and week 16 (T2), using standardized methods and questionnaires, with a follow-up assessment (T2) at university campus to determine whether the observed benefits can be sustained over a longer period. The trial ended according to the planned project timeline, with all participants (150 dyads) completing the intervention and follow-up stages.

### 2.6. Statistical Analysis

Statistical analyses were conducted using IBM-SPSS version 22. The demographic data of the participants and informal caregivers was characterized using descriptive statistics, specifically frequency percentages and mean values with standard deviations.

To address baseline differences between groups, primary effect estimates will be obtained from models that adjust for the baseline value of the outcome and prespecified covariates (list: e.g., age, sex, baseline pain self-efficacy, baseline ADL if imbalanced). Intervention effects will be presented as adjusted between-group differences in change from baseline (or adjusted post-intervention differences with baseline covariate adjustment), with 95% confidence intervals, thereby reducing the risk that observed effects reflect pre-intervention imbalance rather than intervention impact.

An intention-to-treat approach was applied, and the distribution of continuous variables was assessed using the Kolmogorov–Smirnov test. To evaluate intervention effects across the three assessment points (baseline (T0), week 8 (T1), and week 16 (T2)) while accounting for clustering by neighborhood elderly center (NEC) and repeated measurements within individuals, we used multilevel regression models for outcomes that met normality assumptions and generalized estimating equation (GEE) models for outcomes that did not. To address baseline imbalances, baseline (T0) values of each outcome were included as covariates in the main multilevel/GEE models, so that between-group effects at follow-up reflect differences in change beyond baseline levels. The primary parameter of interest was the group effect at follow-up (with time and, where appropriate, a group × time term to estimate differential change over time). Effect sizes were quantified using Cohen’s d for all outcomes, and statistical significance was set at *p* < 0.05. For any missing data, an intention-to-treat analysis was performed.

Although cluster randomization may result in chance baseline imbalances, the primary intervention effects were evaluated using longitudinal models (multilevel regression or GEE, as appropriate) fitted across all measurement occasions (T0, T1, T2). These models included terms for group, time, and the group × time interaction, and the intervention effect was interpreted from the interaction (i.e., differential change over time between groups), thereby accounting for baseline levels even when baseline means differed (e.g., pain self-efficacy and activities of daily living). Clustering and repeated observations were handled by the multilevel/GEE framework, which accounts for within-participant and within-cluster correlations.

### 2.7. Primary Outcome

1. Pain intensity: The Chinese adaptation of the Brief Pain Inventory evaluated the complex aspects of pain, taking into account its intensity and inference with daily activities over the past 24 h. This served as the predetermined outcome measure for facilitating the expansion of the project.

Secondary Outcome:

2. Pain self-efficacy: The Chinese adaption of the Pain Self-Efficacy Questionnaire measured an individual’s belief in their ability to manage activities in spite of pain [27]. It was composed of 10 items regarding a person’s assurance in carrying out 10 tasks or activities while in pain. A higher score signified a more robust belief in self-efficacy.

3. Caregiver Burden Inventory (for informal caregivers only): The Caregiver Burden Inventory consisted of 24 elements that evaluated five aspects of burden associated with the role of caregiving. These are: (1) time dependence; (2) developmental burden; (3) physical burden; (4) social burden; and (5) emotional burden. Participants were asked how often each statement describes their feelings on a scale ranging from 0 (never) to 4 (nearly always). Alpha coefficients for each subscale were satisfactory, ranged from 0.74 to 0.88, and the overall internal consistency was a = 0.91 [28].

4. Depression, Anxiety, and Stress: The Depression Anxiety Stress Scales 21-items (DASS-21), a self-administered psychological instrument to evaluate degrees of depression, anxiety, and stress, was used [29]. Every part has seven items on a 4-point Likert scale ranging from 0 to 3. The Cronbach’s α was 0.912 and the test–retest Pearson correlation coefficient was 0.751 [29]. The variations in the levels of depression, anxiety, and stress among elderly individuals were assessed. A decrease in scores signified a reduction in the levels of depression, anxiety, and stress.

5. Activities of daily living: The Barthel Index, which includes 10 items related to activities of daily living (ADL) such as mobility and self-care ability, was used to measure ADL [30]. These activities, which individuals carry out daily for their overall well-being and self-care, encompass tasks like eating, grooming, bathing, and dressing.

6. Pain knowledge: An 11-item questionnaire was designed to evaluate the participants’ understanding of pain management. The questionnaire included questions such as: Is exercise effective in pain management?’’, ‘‘Can Paracetamol be used to treat fever and pain?’’, ‘‘Is it appropriate to apply a hot or cold compress when sleeping?’’, ‘‘Should deep breathing exercises be used to let the body relax before music therapy?’’. The total score was determined by tallying the correctly answered questions, with higher scores suggesting a more comprehensive understanding of pain management.

### 2.8. Ethical Considerations

All experiments involving human participants and human tissue samples were conducted in accordance with relevant ethical guidelines and regulations. The study protocol was approved by the institutional review board of the Hong Kong Metropolitan University before the start of this study (reference number: HE-HMRF2022/01, 2 April 2022). Informed consent was obtained from all participants and/or their legal guardians prior to inclusion in the study.

## 3. Results

### 3.1. Recruitment Rates

In the first three months of the first year, the research team contacted 20 NECs to establish partnerships. From the fourth month onwards, subject recruitment was carried out, with 30% of subject recruitment (45 dyads) completed by the end of first year. By the end of second year, 75% of subject recruitment (112 dyads) was completed. Subject recruitment was completed (100%; 150 dyads) by the middle of the third year.

### 3.2. Characteristics of Recruited Participants

The demographic characteristics of older adults are displayed in Table 1. There were 150 older adults randomly selected into the experimental group (*n* = 75) and control group (*n* = 75). The majority of the participants were community-dwelling older adults (mean age = 65.44 ± 10.28) with social disadvantages, particularly in aspects of employment and monthly income. The number of female participants (59%) exceeded that of male participants (41%) in both groups combined. Most of them resigned with a spouse and/or a son or daughter. For details of caregivers’ demographic characteristics, please see Table 1.

### 3.3. Physical Outcomes of Older Adults from Baseline to Follow-Up

The pain situation is displayed in Table 2. Compared the experimental group to the control group at baseline, there were no significant differences in pain intensity (*p* = 0.992) and pain interference (*p* = 0.263). At baseline, the experimental and control groups differed significantly in pain self-efficacy and activities of daily living (ADL), indicating imbalance prior to the intervention. Therefore, intervention effects were interpreted based on between-group differences in change over follow-up (i.e., net change from baseline) rather than baseline comparisons alone.

At follow-up (T2), statistically significant between-group differences were observed for pain self-efficacy (*p* < 0.001), pain intensity (*p* = 0.003), and pain interference (*p* < 0.001). Pain self-efficacy increased in both groups (experimental: 4.28 ± 1.45 to 4.89 ± 0.92; control: 3.77 ± 1.39 to 3.96 ± 1.47), whereas pain intensity decreased more in the experimental group (3.66 ± 1.84 to 2.17 ± 1.51) than in the control group (3.66 ± 2.04 to 3.22 ± 2.29). Pain interference declined significantly in the experimental group (2.71 ± 3.13 to 1.46 ± 0.99, *p* < 0.001), supporting an intervention-related reduction over time. Pain knowledge scores increased slightly in the experimental group (7.28 ± 3.70 to 7.43 ± 1.72), but this change was not statistically significant and is reported as a non-significant trend.

Specifically, pain self-efficacy both increased in the experimental (from 4.28 ± 1.45 to 4.89 ± 0.92) and control groups (from 3.77 ± 1.39 to 3.96 ± 1.47). The mean of pain intensity in the experimental group dropped after the intervention period (from 3.66 ± 1.84 at baseline to 2.17 ± 1.51 at T2). Interestingly, the mean of pain intensity in the control group also reduced (from 3.66 ± 2.04 at baseline to 3.22 ± 2.29 at T2), but the decrease was relatively small. For pain interference, the experimental group showed a statistically significant reduction from baseline to T2 (from 2.71 ± 3.13 at baseline to 1.46 ± 0.99 at T2, *p* < 0.001). In contrast, pain knowledge scores in the experimental group increased slightly from 7.28 ± 3.70 at baseline to 7.43 ± 1.72 at T2, but this change did not reach statistical significance.

For physical functioning, ADL scores were significantly different between groups at baseline (*p* = 0.002); thus, the intervention effect was evaluated in terms of change over time and follow-up differences rather than baseline levels. ADL increased significantly within the experimental group over time (*p* = 0.030), and the between-group difference remained significant at the end of the program (*p* = 0.001), suggesting more favorable follow-up ADL outcomes in the experimental group despite the initial imbalance.

### 3.4. Psychological Outcomes of Older Adult and Informal Caregivers from Baseline to Follow-Up

The outcome of psychological health is presented in Table 3. The intervention led to a notable reduction in depression, anxiety, and stress (all of them had *p* < 0.001) in the experimental group, while the control group did not exhibit any significant changes (*p* > 0.05). When comparing the two groups, the differences in depression (*p* = 0.004), anxiety (*p* = 0.010), and stress (*p* = 0.021) were significant.

As depicted in Table 4, the mean of the total caregiver burden in the experimental group significantly reduced from 3.28 ± 5.69 at baseline to 1.70 ± 2.08 at T2 (*p* = 0.008). In comparison, the control group did not exhibit a significant change (*p* = 0.658). The differences between the groups post-intervention were found to be significant (*p* = 0.001).

Effect sizes (Cohen’s d) were calculated to quantify the magnitude of intervention effects on key outcomes at the 16-week follow-up (T2), revealing medium-to-large improvements in the experimental group relative to the control. For pain intensity, the between-group difference in change was −1.05 (95% CI: −1.72 to −0.38), with a Cohen’s d of 0.54, indicating a moderate effect; this exceeds the minimally important difference (MID) of 1 point on the 0–10 Numeric Rating Scale, suggesting clinically meaningful pain reduction for older adults with chronic pain. Pain self-efficacy showed a between-group difference in change of 0.93 (95% CI: 0.52 to 1.34), with d = 0.72 (medium-large effect), surpassing the MID of approximately 0.5 points on the averaged scale (equivalent to ~5 points on the full 0–60 Pain Self-Efficacy Questionnaire), reflecting enhanced confidence in managing pain despite symptoms. For psychological outcomes, depression exhibited a between-group difference of −4.25 (95% CI: −7.12 to −1.38), d = 0.48 (moderate); anxiety −4.05 (95% CI: −7.11 to −0.99), d = 0.43 (moderate); and stress −4.12 (95% CI: −7.59 to −0.65), d = 0.38 (small-moderate), with changes in depression and anxiety meeting or approaching MIDs of 3–5 points on the DASS-21 subscales, indicating clinically relevant reductions in psychological distress. Caregiver burden demonstrated a between-group difference of −2.16 (95% CI: −3.42 to −0.90), d = 0.51 (moderate), exceeding the MID of ~2 points on the total score, underscoring substantial relief in caregiving demands. These effect sizes and clinical thresholds highlight the practical significance of the DPM program beyond statistical significance.

## 4. Discussion

### 4.1. Principal Results

This study examined outcomes associated with a digitally supported, dyadic pain management program (DPM) for community-dwelling older adults and their informal caregivers with chronic non-cancer pain. The program combined weekly pain-management lessons with triweekly WhatsApp reminders and check-ins to support adherence to 30 min exercise practice. Overall, participation in the DPM program was associated with more favorable changes in pain intensity, pain self-efficacy, pain interference, activities of daily living, and psychological health indicators (depression, anxiety, and stress) compared with the control condition; however, these findings should be interpreted cautiously in light of the unblinded design and baseline between-group differences.

### 4.2. Comparison with Prior Work

By using a combined intervention of in-person lessons and WhatsApp group chat, this study demonstrated the pain situation was relieved after the intervention period. Pain intensity in the experimental group significantly declined. In this study, ADL (e.g., self-care and mobility) appeared to improve in the experimental group over follow-up; however, because ADL differed significantly between groups at baseline, the magnitude of post-intervention between-group differences may partly reflect these initial imbalances rather than intervention effects alone. Similarly, pain self-efficacy also differed at baseline, and therefore changes observed at follow-up should be interpreted in terms of baseline-adjusted change (i.e., change from baseline) rather than absolute follow-up values. Consistent with prior evidence that lower pain levels may facilitate greater engagement in daily activities, reductions in pain intensity and interference in the experimental group may have contributed to improved functional participation, but this interpretation remains cautious given the baseline differences and unblinded nature of the study. Evidence from mHealth pain intervention studies also suggests that hybrid delivery (in-person support plus remote access) can be associated with improved pain outcomes, and the combined on-campus sessions and WhatsApp support in the current program may have helped participants access timely guidance and clarify misunderstandings. Nevertheless, further research with stronger control of baseline imbalances (and, where feasible, improved blinding) is needed to confirm the net impact of the program and to determine how pain self-management gains can be sustained over the longer term for both older adults and informal caregivers.

Attaining sufficient pain control is challenging, especially for older adults, due to the related use of multiple medications and lack of access to pain management information, as many of them tend to rely on sources like friends and relatives [31]. A WhatsApp group chat was created as part of the intervention in this study to aim to increase participants’ pain knowledge through active interactions. The current results show that their pain knowledge was strengthened. This finding aligned with a that having a shared space for communication between information providers and older adults is crucial in obtaining health information and practicing disease or pain self-management [32]. This support group chat provided participants with a reliable source of information and more confidence to manage their pain, as the pain self-efficacy improved significantly in the experimental group. This delivery method positively influenced participants’ behavior. Participants could easily spread the knowledge learned in this program to their friends and families by simply clicking the WhatsApp “forward” button.

This present study shows that the psychological health in the experimental group improved after the study period, with significant reductions in depression, anxiety, and stress. This result aligns with the Bostrøm et al. study, which revealed that an evidence-informed digital pain self-management program substantially enhanced psychological well-being by lessening depression symptoms and boosting the ability to control thoughts, emotions, and actions in individuals suffering from chronic pain [33]. A meta-analysis of 19 randomized controlled trials also found that text messaging-based health promotion interventions led to positive behavioral changes [34]. When participants were well-supported with clear instructions, adequate and accurate information, and a friendly digital environment, participants’ psychological distress substantially diminished.

Finally, the DPM program lowered informal caregivers’ level of burden significantly in the experimental group. This result aligns with a newly published scoping review of 9 studies on online support groups for family caregivers, which reported that caregivers expressed satisfaction with the informational and emotional support received and found these groups beneficial [35]. Therefore, a balanced approach of web-based group activities and socially distanced in-person activities is helpful. The DPM program educated participants with pain self-management skills and exercises and gave the informal caregivers a supportive platform to ask questions and address concerns. These forms of support further advanced the informal caregivers’ ability to assist older adults in pain management. Previous studies also marked the importance of online support groups on caregiver burden in caring for older adults [36,37,38]. The needs of each informal caregiver may vary. Thus, providing these caregivers with multimodal support decreased their overall burden.

### 4.3. Limitations

First, this study was conducted during the COVID-19 pandemic, and strict social distancing measures may have influenced participants’ behaviors and outcomes, limiting generalizability to pre- and post-pandemic contexts. Second, key outcomes were self-reported, and some questionnaires were completed with assistance from informal caregivers due to potential comprehension or writing difficulties among older adults; therefore, reporting bias cannot be excluded. Third, despite randomization, statistically significant baseline imbalances were observed (e.g., pain self-efficacy and activities of daily living), which may have influenced post-intervention between-group comparisons and should be considered when interpreting the net intervention effect. Fourth, true participant blinding was not feasible given the nature of the program (in-person sessions and WhatsApp-based support), and awareness of group allocation may have contributed to expectancy or performance effects. Finally, because participation required a smartphone with internet connectivity and engagement with WhatsApp-based activities, the sample may have over-represented individuals with higher digital literacy or greater comfort with technology, introducing potential selection bias and limiting applicability to older adults with lower digital access.

## 5. Conclusions

This study examined outcomes associated with a multicomponent pain management program that combined in-person and digital delivery for older adults with chronic pain and their informal caregivers. Overall, the findings indicate that participation in the DPM program was associated with improvements in several pain- and well-being-related outcomes and with lower caregiver burden relative to the control group; however, these results should be interpreted cautiously given the lack of blinding and the presence of baseline between-group differences. The combined delivery approach may offer a feasible way to provide ongoing support for pain self-management, but further well-controlled studies are needed to confirm effectiveness and clarify which components drive the observed changes. With appropriate implementation and evaluation, the program may inform future evidence-based nursing practice and home-based interventions for older adults and caregivers.

### Trial Protocol

The full trial protocol named, “A Dyadic Pain Management Program for Community-Dwelling Older Adults with Chronic Pain: Study Protocol for a Cluster Randomized Controlled Trial,” can be accessed using DOI: 10.3390/ijerph191912186 [17].

## Figures and Tables

**Table 1:** Demographic characteristic of the 150 older adults with chronic pain enrolled in the Dyadic Pain Management program (2022–2023).

Demographic Data	Overall (*n* = 150)		Experimental Group (*n* = 75)		Control Group (*n* = 75)		*p* Value
Age (Mean ± SD) Range	65.44 ± 10.28 (50–97)		65.49 ± 9.91 (50–89)		65.39 ± 10.71 (50–97)		0.95
	*n*	%	*n*	%	*n*	%	
***Gender***							-
Female	88	59	49	65	39	52	
Male	62	41	26	35	36	48	
***Marital status***							*0.608*
Single	2	1.3	1	1.3	1	1.3	
Married/partnered	117	78	55	73	62	83	
Divorced	8	5.3	5	6.7	3	4	
Widowed	23	15	14	19	9	12	
***Highest education level***							*0.352*
No formal education	11	7.3	7	9.3	4	5.3	
Primary school	46	31	22	29	24	32	
Middle school	66	44	36	48	30	40	
College degree or above	27	18	10	13	17	23	
***Employment***							*0.099*
Unemployed	4	2.7	0	0	4	5.3	
Employed	52	35	24	32	28	37	
Employed (Part-time)	13	8.7	9	12	4	5.3	
Retired	81	54	42	56	39	52	
***Monthly income*** *(HKD$)*							*0.345*
<10,000	61	41	34	45	27	36	
10,000–14,999	19	13	9	12	18	24	
15,000–19,999	23	15	12	16	11	15	
20,000–24,999	15	10	7	9.3	8	11	
25,000–29,999	5	3.3	4	5.3	1	1.3	
>30,000	27	18	9	12	18	24	
***Living Arrangement***							*0.031*
Alone	15	10	9	12	6	8	
With spouse *(1)*	49	31	26	35	20	27	
With son or daughter *(2)*	43	29	24	32	19	25	
With domestic helper *(3)*	3	2	2	2.7	1	1.3	
(1) and (2)	38	25.3	13	17.3	25	33.3	
(1) and (3)	1	0.7	1	1.3	0	0	
(2) and (3)	1	0.7	0	0	1	1.3	
(1), (2) and (3)	2	1.3	0	0	2	2.7	
With relative(s)	1	0.7	0	0	1	1.3	

**Table 2:** Pain situations and physical health outcomes of the 150 older adults with chronic pain enrolled in the Dyadic Pain Management program (2022–2023) ^a–c^.

Categories (Range)		Experimental (*n* = 75)		Control (*n* = 75)		Between-Group *p* Value
		Mean ± SD	Within *p*	Mean ± SD	Within *p*	
Pain Self-Efficacy (0–10)	T0	4.28 ± 1.45		3.77 ± 1.39		0.047 ^a^
	T1	4.57 ± 1.00	0.023 ^a^	3.97 ± 1.53	0.090	0.013 ^a^
	T2	4.89 ± 0.92	0.000 ^a^	3.96 ± 1.47	0.387	0.000 ^a^
Pain Intensity (0–10)	T0	3.66 ± 1.84		3.66 ± 2.04		0.992
	T1	2.87 ± 1.54	0.000 ^a^	3.33 ± 2.09	0.008	0.138
	T2	2.17 ± 1.51	0.000 ^a^	3.22 ± 2.29	0.019	0.003 ^a^
Pain Interference (0–10)	T0	2.71 ± 3.13		3.19 ± 2.10		0.263
	T1	2.21 ± 1.53	0.182	2.66 ± 2.29	0.002	0.174
	T2	1.46 ± 0.99	0.000 ^a^	3.96 ± 1.47	0.086	0.000 ^a^
Pain Knowledge (0–11)	T0	7.28 ± 3.70		7.81 ± 1.99		0.226
	T1	7.34 ± 1.75	0.969	7.71 ± 2.98	0.900	0.288
	T2	7.43 ± 1.72	0.917	8.06 ± 3.11	0.650	0.137
Activities of Daily Living (0–100)	T0	98.53 ± 4.82		91.33 ± 20.01		0.002 ^a^
	T1	99.38 ± 2.45	0.118	92.27 ± 19.14	0.631	0.003 ^a^
	T2	99.84 ± 0.88	0.030 ^a^	92.80 ± 17.79	0.346	0.001 ^a^

^a^*p* value should be less than 0.05 to be considered as significant. ^b^ Remarks: T0: Before the intervention; T1: 8-week follow-up; T2: 16-week follow-up; ^c^ Between-group *p* values at follow-up (T1, T2) are from baseline-adjusted longitudinal multilevel regression models (for normally distributed outcomes) or GEE models (for non-normal outcomes), accounting for clustering by neighborhood elderly center (NEC) and repeated measures; baseline (T0) value of the outcome was included as a covariate. ‘Within *p*’ values represent within-group change over time from the same longitudinal model.

**Table 3:** Psychological health outcomes of the 150 older adults with chronic pain enrolled in the Dyadic Pain Management program (2022–2023) ^a–c^.

**Categories (Range)**		**Experimental** **(*n* = 75)**		**Control** **(*n* = 75)**		**Between-Group *p* Value**
		**Mean ± SD**	**Within *p***	**Mean ± SD**	**Within *p***	
**Depression** **(0–36)**	T0	8.24 ± 9.83		8.37 ± 8.81		0.922
	T1	5.84 ± 7.59	0.001 ^a^	7.21 ± 9.91	0.117	0.323
	T2	3.05 ± 4.85	0.000 ^a^	7.30 ± 11.27	0.243	0.004 ^a^
**Anxiety** **(0–36)**	T0	8.67 ± 10.24		8.64 ± 11.75		0.985
	T1	5.84 ± 7.62	0.001 ^a^	7.27 ± 10.56	0.040	0.351
	T2	3.56 ± 5.52	0.000 ^a^	7.61 ± 11.98	0.337	0.010 ^a^
**Stress** **(0–40)**	T0	12.43 ± 14.55		11.28 ± 9.97		0.484
	T1	9.34 ± 9.13	0.010 ^a^	10.18 ± 11.88	0.261	0.601
	T2	6.03 ± 6.58	0.000 ^a^	10.15 ± 13.52	0.368	0.021 ^a^

^a^*p* value should be less than 0.05 to be considered as significant. ^b^ Remarks: T0: Before the intervention; T1: 8-week follow-up; T2: 16-week follow-up; ^c^ Between-group *p* values at follow-up (T1, T2) are from baseline-adjusted longitudinal multilevel regression models (for normally distributed outcomes) or GEE models (for non-normal outcomes), accounting for clustering by neighborhood elderly center (NEC) and repeated measures; baseline (T0) value of the outcome was included as a covariate. ‘Within *p*’ values represent within-group change over time from the same longitudinal model.

**Table 4:** The caregiver burden inventory outcomes of the 150 informal caregivers with chronic pain enrolled in the Dyadic Pain Management program (2022–2023) ^a–c.^

Categories (Range)		Experimental (*n* = 75)		Control (*n* = 75)		Between-Group *p* Value
		Mean ± SD	Within *p*	Mean ± SD	Within *p*	
Total: The Caregiver Burden Inventory (0–16)	T0	3.28 ± 5.69		4.18 ± 4.02		0.218
	T1	2.76 ± 3.26	0.349	3.71 ± 4.85	0.151	0.137
	T2	1.70 ± 2.08	0.008 ^a^	3.86 ± 5.49	0.658	0.001 ^a^
Subcategories: Development (0–4)	T0	0.62 ± 1.22		0.81 ± 0.83		0.245
	T1	0.50 ± 0.72	0.275	0.68 ± 1.11	0.181	0.230
	T2	0.28 ± 0.52	0.007 ^a^	0.73 ± 1.19	0.642	0.003 ^a^
Physical (0–4)	T0	0.83 ± 1.58		0.91 ± 1.07		0.621
	T1	0.67 ± 0.83	0.311	0.83 ± 1.21	0.273	0.310
	T2	0.46 ± 0.62	0.032 ^a^	0.85 ± 1.28	0.664	0.015 ^a^
Emotional (0–3)	T0	0.32 ± 0.77		0.51 ± 0.76		0.082
	T1	0.32 ± 0.59	0.993	0.49 ± 0.93	0.894	0.172
	T2	0.13 ± 0.33	0.037 ^a^	0.52 ± 0.95	0.997	0.001 ^a^
Social (0–3)	T0	0.58 ± 1.03		0.70 ± 0.85		0.415
	T1	0.49 ± 0.69	0.408	0.59 ± 0.97	0.092	0.457
	T2	0.30 ± 0.51	0.009 ^a^	0.68 ± 1.12	0.957	0.007 ^a^
Time (0–4)	T0	0.93 ± 1.38		1.23 ± 0.94		0.116
	T1	0.78 ± 0.77	0.289	1.13 ± 1.03	0.149	0.023 ^a^
	T2	0.53 ± 0.49	0.006 ^a^	1.08 ± 1.24	0.230	0.000 ^a^
Intensity (0–9)	T0	3.66 ± 1.84		3.66 ± 2.04		0.992
	T1	2.87 ± 1.54	0.000 ^a^	3.33 ± 2.09	0.008 ^a^	0.138
	T2	2.17 ± 1.51	0.000 ^a^	3.22 ± 2.29	0.019 ^a^	0.003 ^a^
Interference (0–10)	T0	2.71 ± 3.13		3.19 ± 2.10		0.263
	T1	2.21 ± 1.53	0.182	2.66 ± 2.29	0.002 ^a^	0.174
	T2	1.46 ± 0.99	0.000 ^a^	2.74 ± 2.75	0.086	0.000 ^a^

^a^*p* value should be less than 0.05 to be considered as significant. ^b^ Remarks: T0: Before the intervention; T1: 8-week follow-up; T2: 16-week follow-up; ^c^ Between-group *p* values at follow-up (T1, T2) are from baseline-adjusted longitudinal multilevel regression models or GEE models (as appropriate), accounting for clustering by NEC and repeated measures; baseline (T0) value of the outcome was included as a covariate. ‘Within *p*’ values represent within-group change over time from the same longitudinal model. Baseline (T0) between-group *p* values are simple unadjusted comparisons.

## Data Availability

The data that support the findings of this study are available from the corresponding author, M.M.Y.T., upon reasonable request because of privacy and ethical restrictions, as they contain sensitive information from older adult participants and their informal caregivers.

## Abbreviations

ADL	activities of daily living
COVID-19	coronavirus disease 2019
DPM	dyadic pain management
IASP	International Association for the Study of Pain
mHealth	mobile health
NECs	neighborhood elderly centers
RCT	randomized controlled trial
